# *GADD45A* is Essential for Granulosa Cells Differentiation and Ovarian Reserve in Human and Mice

**DOI:** 10.1111/jcmm.70820

**Published:** 2025-09-07

**Authors:** Juncen Guo, Yuanyuan Hu, Qi Cao, Ying Zhang, Yihe Jia, Lan Liu, Yanru Zeng, Xiao Wu, Yuelin Song, Maosen Yang, Wenming Xu, Yang Hu, Wei Huang, Tian Tang

**Affiliations:** ^1^ Department of Obstetrics and Gynecology, West China Second University Hospital Sichuan University Chengdu China; ^2^ Key Laboratory of Birth Defects and Related Diseases of Women and Children (Sichuan University) Ministry of Education Chengdu China; ^3^ Joint Laboratory of Reproductive Medicine, SCU‐CUHK, Key Laboratory of Obstetric, Gynaecologic and Paediatric Diseases and Birth Defects of Ministry of Education, West China Second University Hospital, Med‐X Centre for Manufacturing Sichuan University Chengdu China; ^4^ Reproductive Medical Center, Department of Obstetrics and Gynecology West China Second University Hospital, Sichuan University Chengdu Sichuan China; ^5^ Bazhong Central Hospital Bazhong Sichuan Province China; ^6^ Neijiang Maternal and Child Health Hospital Neijiang Sichuan Province China; ^7^ College of Biomedical Engineering and National Engineering Research Center for Biomaterials Sichuan University Chengdu China

**Keywords:** DOR, GADD45A, granulosa cells, mouse model, ovary

## Abstract

Diminished ovarian reserve (DOR) poses significant challenges in reproductive health, with emerging evidence implicating DNA damage repair pathways. While GADD45A is a critical regulator of DNA repair, cell cycle and apoptosis, its role in DOR pathogenesis remains unexplored. We employed transcriptome sequencing, qPCR and Western Blot analyses to compare *GADD45A* expression in granulosa cells (GCs) between DOR patients and controls. Functional studies included *GADD45A* overexpression/knockdown in human granulosa cells (KGN line) and phenotypic characterisation of *Gadd45a* knockout (KO) mice. Ovarian reserve parameters (follicle counts, hormone levels, oestrus cyclicity) and in vitro fertilisation outcomes were systematically evaluated. DOR patients exhibited significant upregulation of GADD45A in GCs, concomitant with reduced FSHR and CYP19A1 expression. In vitro experiments revealed that *GADD45A* overexpression disrupted both proliferation (Cell cycle analysis and EdU staining) and differentiation (Reduced CYP19A1 and FSHR expression) in GCs, while knockdown specifically impaired differentiation (Elevated CYP19A1 and FSHR expression). *Gadd45a* KO mice displayed hallmark DOR features: irregular oestrus cycles (Shorter oestrus), ovarian volume reduction, ovarian hormones dysregulation and decreased ovarian reserve (reduced primordial follicles and antral follicles, and increased atretic follicles). We found GADD45A was robustly expressed in the ovarian stroma and GCs of atretic follicles. KO oocytes showed compromised developmental competence with decreased two‐cell embryo rate in vitro fertilisation. Our findings establish *GADD45A* dysregulation as a mechanistic contributor to DOR through dual impacts on granulosa cell differentiation and follicle survival. The *Gadd45a* KO mouse recapitulates key clinical DOR phenotypes, providing a validated model for therapeutic discovery.

## 1 Background

Diminished ovarian reserve (DOR) is a disorder of ovarian function characterised by a decreased number of oocytes and/or impaired oocyte quality with advanced age. Some women experience DOR much earlier and become prematurely infertile; this condition is known as pathologic DOR and develops into premature ovarian failure (POF) [1, 2]. The US‐based National Society for Assisted Reproductive Technology (SART) states that 32% of in vitro fertilisation (IVF) cycles (approximately 66,000 cycles) carry a diagnosis of DOR, which contributes significantly to poor response in ovarian stimulation [3]. Ovarian reserve is a complex phenomenon associated with genetic, environmental, autoimmune, idiopathic, iatrogenic factors and age [1, 4, 5]. While these multifactorial aetiologies highlight the complexity of DOR pathogenesis, the lack of definitive biomarkers and targeted therapies underscores the urgent need to identify molecular drivers of ovarian dysfunction.

With the development of sequencing methods, including submicroscopic copy number variations (CNVs), single nucleotide polymorphisms (SNPs), genome‐wide association studies (GWAS) and whole‐exome sequencing (WES), a few candidate genes related to ovarian phenotype have been identified [4, 6]. However, few variants corresponding to human orthologues have been confirmed in DOR patients, possibly due to the small size of the cohorts or the ethnicity of the groups investigated, and until now, only a few genes (such as *FMR1* premutation, *BMP15, GDF9*, *FSHR* and *BRCA*) have been acknowledged as diagnostic biomarkers [4, 6, 7]. Noticeably, genes engaging in DNA damage repair pathways may play a crucial role in the cause of POF. Accumulating laboratory, translational and clinical evidence has emerged within the past decades indicating the role of BRCA function in homologous DNA recombination and ataxia telangiectasia mutated (ATM)‐mediated DNA double‐strand break (DSB) repair in ovarian aging [8, 9]. When DNA damage is not repaired, especially in nondividing or slowly dividing cells, where unrepaired damage tends to accumulate over time, cells might be eliminated by apoptotic cell death or undergo senescence to avoid severe mutagenic consequences. Eventually, follicles, which are mostly in a resting state from birth, might undergo accelerated apoptosis and present DOR, even the POF phenotypes [6, 7, 10].

Therefore, this study aims to discover novel DOR susceptibility genes through systematic analysis of BRCA1‐interacting partners in DNA damage response networks. The growth arrest and DNA damage‐inducible 45 alpha (*GADD45A*) gene plays a pivotal role in the regulation of cellular reactions responsible for stress as its transcript levels increase in response to stressful growth arrest conditions and DNA damage repair [11, 12, 13]. When DNA damage is present, *GADD45A* can be activated by BRCA1 (Breast cancer susceptibility gene 1) via recruitment of p53, CDK1 (cyclin dependent kinase 1), cyclin B1, and other targeting transcription factors [12, 14]. The level of GADD45A is related to the cell cycle and is highest in the G1 phase, with a substantial reduction in S. Moreover, emerging evidence has proved that GADD45A, which is highly associated with DNA repair and apoptosis in follicles of sows and cows, could be a potential marker of the quality of follicles, especially atresia follicles [15, 16, 17]. However, the impact of GADD45A on human reproduction, especially on ovarian function, remains unexplored.

Granulosa cells (GCs) encompass the oocytes and serve vital functions in follicle development and follicular atresia [18]. These cells undertake functions by not only protecting and interacting with oocytes but also through steroidogenesis and endocrine functions [19]. Granulosa cell differentiation in the ovary is a complex process that involves the development and maturation of granulosa cells, which are essential for the production of oestrogen and the support of oocyte growth and development. Abnormal differentiation in GCs has been found to impair ovarian function and to be related to human reproductive disorders, such as premature ovarian insufficiency (POI) [20, 21]. This differentiation process is tightly regulated by various factors, including hormones, growth factors and signalling pathways [22]. During differentiation, granulosa cells undergo changes in gene expression, morphology and function, ultimately leading to the formation of mature granulosa cells that can support oocyte development and ovulation. Although several factors have been shown to be involved in GC differentiation, the regulatory mechanisms remain largely elusive.

In this study, using granulosa cell samples from DOR patients, we conducted a transcriptome study, and our results showed that *GADD45A* was dysregulated in the GCs of DOR. Furthermore, we proved that the imbalance of *GADD45A* expression could impair GC differentiation. We also found that the *Gadd45a* knockout mouse model exhibited phenotypes of diminished ovarian reserve. Our findings provide the first evidence for the role of *GADD45A* in GC differentiation and suggest that dysregulated *GADD45A* resulting in dysfunctional GC differentiation may be involved in the pathogenesis of DOR. Also, our findings might provide an ideal DOR mouse model for further investigation and a promising targeted treatment in clinical practice.

## 2 Methods

### 2.1 Study Populations

The participants were divided into two groups (listed in Table S1): DOR group (*n* = 18) and Control group (*n* = 16). We included age, nationality, body mass index (BMI) matching and normal ovarian function women who undergo IVF treatment due to male or oviduct factors as controls. Granulosa cells were collected from women undergoing treatment with assisted reproduction technologies for ovarian infertility after informed consent was obtained, and the study was approved by the Ethics Committee of West China Second University Hospital of Sichuan University (2020096). According to the Practice Committee of the American Society for Reproductive Medicine (ASRM), there is “no uniformly accepted definition of DOR”. DOR is diagnosed when ovarian reserve testing indicates abnormal findings including low antral follicular count (AFC, < 7 follicles) and low anti‐Müllerian hormone (AMH, < 1.1 ng/mL) [1, 3]. Exclusion criteria: Previous pelvic or ovarian surgery history, pelvic tumour radiotherapy or chemotherapy history; autoimmune diseases such as systemic lupus erythematosus, Hashimoto's thyroiditis, psoriasis, etc.; family history of genetic diseases; intrauterine adhesions, submucosal fibroids, hydrosalpinx, uterine malformations (unicornuate uterus, bicornuate uterus, etc.); repeated miscarriage and repeated implantation failure history; endometriosis and adenomyosis, etc.

Characteristics of patients were obtained from their electronic medical records (EMR) at West China Second University Hospital of Sichuan University. Basal hormones were measured on the second day of the menstrual cycle in the laboratory of West China Second University Hospital of Sichuan University by chemiluminescence (Siemens ADVIA Centaur CP, Siemens Medical Solutions Diagnostics, Tarrytown, NY, USA). AFC was evaluated by two independent experienced clinicians. Written informed consent was obtained from all patients.

### 2.2 Isolation of Human Granulosa Cell

After obtaining the cumulus‐oocyte complexes, the cumulus granulosa cells enveloping the oocytes were mechanically stripped from patients with diminished ovarian reserve as well as patients with normal ovarian reserve. The suspension containing granulosa cells was centrifuged at 300 g for 10 min. Resuspended the pellet in 1 mL of PBS and slowly overlaid with an equal volume of 50% Percoll (Solarbio, P8370, Beijing, China). The samples were centrifuged at 300 g for 20 min, aspirated the intermediate cell layer and finally washed three times with PBS. The final cell precipitations were collected for subsequent detections.

### 2.3 Whole Transcriptome Sequencing and Bioinformatic Analysis

The total RNA from the granulosa cells or ovaries was extracted with TRIzol Reagent (Life Technologies, Carlsbad, California, USA) using RQ1 RNase‐free DNase (Promega, Madison, USA) to remove the genomic DNA. mRNA was purified from total RNA using poly‐T oligo‐attached magnetic beads (Invitrogen, Waltham, Massachusetts, USA). Double‐stranded complementary DNAs were synthesised with Superscript II reverse transcriptase (Invitrogen, Waltham, Massachusetts, USA) and random hexamer primers. The cDNAs were then fragmented by nebulization, and the standard Illumina protocol was performed thereafter to establish the mRNA‐seq library. For data analysis, base calling was performed using CASAVA. Reads were aligned to a genome using the split read aligners TopHat and Bowtie2 with default parameters. HTSeq was used for estimating abundances. |log_2_FC| > 1 and a *P*‐value cut‐off of 0.05 were considered statistically significant in the analysis of differentially expressed genes (DEGs). The DEG (DOR vs. Control) was examined by functional enrichment analysis. The pathway enrichment for the DEGs was performed via the BGI Multiomics system. KEGG pathways with *Q*‐value ≤ 0.05 were significantly enriched.

### 2.4 Cell Culture and Transfection

The steroidogenic GC‐like tumour cell line (KGN) was cultured with DMEM/F12 (HyClone, SH30023.01, Logan, Utah, USA) containing 10% FBS (HyClone, SH30070.03, Logan, Utah, USA) and 1% penicillin–streptomycin (HyClone, SV30010, Logan, Utah, USA) in a humidified incubator containing 5% CO_2_ at 37°C. SiRNAs targeting *GADD45A* mRNA and overexpressed lentivirus were purchased from OBiO Tech (Shanghai, China). Sequences of SiRNA were listed in Table S2. Cells (2 × 10^5^) in 6‐well plates were transfected with 2.5 nM SiRNA using transfection reagent (jetPRIME transfection reagent) (Assen, Netherlands). For establishing overexpressed GADD45A KGN cells in vitro, the virus concentration of MOI = 10 was used for lentivirus to infect KGN cells.

### 2.5 Cell Cycle Analysis

To detect the cell cycle of overexpressed, knockdown and control KGN, we followed the instructions of the Cell Cycle Analysis Kit (Beyotime, C1052, Shanghai, China). Cells were harvested and fixed in 75% ethanol for 2 h at 4°C and then stained with a solution containing propidium iodide (0.05 mg/mL), RNase A (1 mg/mL), and 0.3% Triton X‐100 in the dark for 30 min. The percentage of cells in different phases of the cell cycle was examined by measuring the DNA content (propidium iodide intensity) with a flow cytometer (BD Biosciences, Franklin Lakes, USA), and populations of G1, S and G2/M phase cells were determined with the ModFIT software. Each experiment was repeated three independent times.

### 2.6 EdU Staining for Detection of Cellular Proliferating Capacity

KGN cells after SiRNA transfection or overexpressed lentivirus infection were plated in 24‐well plates for the EdU assay using a BeyoClick EdU Cell Proliferation Kit with Alexa Fluor 594 (Beyotime, C0078S, Shanghai, China) following the manufacturer's instructions. Subsequently, cells were incubated with EdU for 2 h. Next, the medium was aspirated and the cells were washed twice with PBS before fixing with paraformaldehyde. Cells were washed thrice with washing buffer (3% BSA in PBS) and permeated with 0.3% Triton X‐100 for a further 15 min. Next, the cells were washed three times with washing buffer, and incubated with the Click Reaction Mixture and Hoechst 33,342 according to the manufacturer's instructions. Cells stained with both red and blue were considered EdU‐positive cells. The cell proliferation ratio was the ratio of EdU‐positive cells to the total cell count. Each experiment was repeated three independent times.

### 2.7 *Gadd45a‐* Knockout (−/−) (KO) Mice

C57BL/6 *Gadd45a*‐heterozygous (+/−) female mice were constructed by means of the CRISPR/Cas9 method (View solid‐biotech, Beijing, China) with deletion of a 2007‐bp fragment encompassing exons 1–4. PCR‐based genotyping analysis with tail genomic DNA was performed for *Gadd45a* using the following primers: 5′‐CTCCGCACCAGAAGGCTCCA‐3′ (forward) and 5′‐GGCTTATTTGAAAGTAACCTGGCC‐3′ (reverse). Subsequently, *Gadd45a*‐heterozygous (+/−) female mice were bred with C57BL/6 wildtype (WT) male mice to produce heterozygous (+/−) male mice. We matched heterozygous parents to produce the female *Gadd45a*‐knockout (−/−) (KO) mice. Then, heterozygous (+/−) female mice and male KO mice were caged together to produce more mutant mice. We compared oestrous cycle, serum hormone levels and ovarian morphology between two groups at the age of 16 weeks. All animal experiments were performed in accordance with the ethical guidelines approved by the Animal Care and Research Committee of Sichuan University (K2019038).

### 2.8 Oestrous Cycle Determination

Oestrous cycles of wildtype group (*n* = 3) or KO group (*n* = 3) were monitored beginning at 16 weeks old. Vaginal cytology was assessed by dipping a sterile swab in water and gently swabbing the outer half of the vaginal canal. The vaginal samples were transferred to a microscope slide, air dried, stained using Wright Stain solution, dehydrated and then coverslipped prior to visualisation with a light microscope. The mouse oestrus cycle was evaluated by observing the relative proportions of epithelial nucleated cells, squamous cells and leucocytes in vaginal smears [23].

### 2.9 Measurement of Hormone Levels

Follicle‐stimulating hormone (FSH) and AMH were applied to evaluate ovarian reserve. Therefore, the levels of follicle‐stimulating hormone (FSH) and anti‐Mullerian hormone (AMH) in serum samples were measured using ELISA kits (Elabscience, Wuhan, China) in both *Gadd45a* KO and WT mice. The characteristics of the AMH ELISA Kit: Sensitivity 0.47 ng/mL, specific for mouse AMH with no significant cross‐reactivity, inter‐ and intra‐assay CVs < 10%. The characteristics of the FSH ELISA Kit: Sensitivity 0.94 ng/mL, specific for mouse FSH with no notable cross‐reactivity. Inter‐ and intra‐assay CVs < 10%. The ELISAs were performed according to the manufacturer's instructions. The estradiol (E2) and testosterone (T) concentrations were measured by radioimmunoassay (Beijing North Institute of Biotechnology, Beijing, China). The characteristics of the Testosterone RIA Kit: Sensitivity 0.02 ng/mL, intra‐assay CV < 10%, inter‐assay CV < 15%. Cross‐reactivity with its analogue: dihydrotestosterone (1.1 × 10^−2^%), estradiol (2.1 × 10^−2^%), estriol (2 × 10^−15^%) and androstenedione (1.2 × 10^−5^%). The characteristics of Estradiol RIA Kit: Sensitivity ≤ 2 pg/mL, intra‐assay CV < 10%, inter‐assay CV < 15%. Cross‐reactivity with its analogue: estriol (0.016%), progesterone (< 0.01%), testosterone (0.01%).

### 2.10 Ovarian Morphometry and Follicle Examination

Ovarian grafts were fixed in 4% buffered paraformaldehyde overnight, embedded in paraffin, serially sectioned at a thickness of 5 μm using a microtome, and stained with haematoxylin. Only follicles with a stained nucleus in the oocyte were counted. The total number of follicles in each section was counted, and the percentages of all types of follicles in the wildtype group (*n* = 3) or KO group (*n* = 3) were calculated. The categories of follicles were as follows: primordial, when it contained an oocyte around a single layer of flat squamous granulosa cells; primary, when it contained an oocyte surrounded by a layer of cuboidal granulosa cells; secondary, when it contained an oocyte surrounded by more than two layers of granulosa cells; and atretic, when a single large space was evident [24].

### 2.11 Protein Extraction and Western Blotting

Tissue lysates were prepared by incubation in RIPA buffer for 30 min on ice followed by centrifugation at 12,000 r.p.m. for 15 min. The protein concentration of the extracts was determined using a Bradford protein assay kit (Thermo Fisher, Waltham, Massachusetts, USA). Equal amounts of protein extracts were separated by 12% SDS‐polyacrylamide gel electrophoresis (SDS‐PAGE) and transferred to 0.45‐μm polyvinylidene difluoride (PVDF) membranes (Thermo Fisher, Waltham, Massachusetts, USA). The membranes were blocked in 5% milk for 1 h at room temperature, then washed and incubated with primary antibodies (GADD45A (sc‐6850, Santa Cruz Biotechnology, Santa Cruz, USA), FSHR (340,416, Zenbio, Chengdu, China), CYP19A1 (R381716, Zenbio, Chengdu, China), GAPDH (A19056, ABclonal, Wuhan, China)) overnight at 4°C. Following overnight incubation, the membranes were washed thrice with 0.1% Tween 20 in Tris‐buffered saline and incubated with HRP‐conjugated goat anti‐rabbit or anti‐mouse IgG secondary antibodies for 1 h at room temperature. Antibody binding was detected using an ECL detection system; protein levels were normalised to GAPDH. Each experiment was repeated three independent times.

### 2.12 Immunofluorescence Staining

For ovarian tissue staining, samples were first fixed in 4% paraformaldehyde. Next, the tissues were placed in ethanol for gradient dehydration and then embedded in paraffin. The samples had been sectioned at a thickness of 5 μm. The sections on the slides were deparaffinised, boiled in 10 mM citrate buffer (pH 6.0) for 10 min and immersed in 3% H_2_O_2_ for 10 min. The slides were washed with 1× PBS, incubated with 10% donkey serum with PBS for blocking and incubated with primary antibody GADD45A (sc‐6850, Santa Cruz Biotechnology, Santa Cruz, USA) in 10% donkey serum at a dilution of 1:50 at 4°C overnight. After being rinsed with 1× PBS three times, the slices were incubated with the secondary antibody in 10% donkey serum: Alexa Fluor 488 1:1000 (A21206; Thermo Fisher Scientific, Waltham, Massachusetts, USA) for 2 h at room temperature. Images were acquired using a laser scanning confocal microscope (Olympus, Shinjuku, Japan).

### 2.13 RNA Isolation and Quantitative Reverse Transcription PCR Analysis

Total RNA was isolated with a Total RNA Kit (Omega Bio‐tek, Norcross, USA) according to the manufacturers' protocol. Then, a Prime Script reverse transcription reagent kit (TaKaRa, Tokyo, Japan) was applied for cDNA synthesis. Real‐time PCR was carried out using SYBR green sequence detection reagents (Takara Tokyo, Japan) in a 20‐μL reaction which contains 1 μL of cDNA, 10 μL of SYBR green, 1 μL of 10 μM primer (each) and 7 μL RNAase‐free water. Primer sequences were as follows:

Mouse *Gadd45a*, 5′‐CGTGCGTTCTGCTGCGAGAA‐3′ (forward) and 5′‐CCTTCCATTGTGATGAATGTGGGTTC‐3′ (reverse).

Human *GADD45A*, 5′‐CTGGAGGAAGTGCTCAGCAAAG‐3′ (forward) and 5′‐AGAGCCACATCTCTGTCGTCGT‐3′ (reverse).

Human *GAPDH*, 5′‐GTCTCCTCTGACTTCAACAGCG‐3′ (forward) and 5′‐ACCACCCTGTTGCTGTAGCCAA‐3′ (reverse).

Mouse *Gapdh*, 5′‐TGCACCACCAACTGCTTAGC‐3′ (forward) and 5′‐GGCATGGACTGTGGTCATGAG‐3′ (reverse).

The PCR scheme was 2 min at 95°C, then 40 cycles of 15 s at 95°C and 60 s at 58°C and was performed on an Applied Biosystems QuantStudio 3 system (Thermo Fisher Scientific, Waltham, Massachusetts, USA). A dissociation curve was performed to identify the specificity of the PCR products. Relative gene expression was calculated with the 2 ΔΔCT method with *GAPDH* as a reference gene.

### 2.14 In Vitro Fertilisation (IVF)

Each group (WT or KO female mice) for In vitro fertilisation has three mice with matching age. Oocyte‐cumulus complexes (OCCs) were collected from the oviductal ampulla of superovulated mice (10 weeks old) 14 h after hCG injection. The OCCs were then placed in a 90 μL drop of TYH medium (Nanjing Aibei Biotechnology, M2030, Nanjing, China) covered with mineral oil. Sperm were capacitated by incubating cauda epididymal of male mice (C57BL6/J, 8–10 weeks old) in 500 μL drops of TYH medium for 1 h with 5% CO_2_ at 37°C. Subsequently, the capacitated sperm suspension was added to the 90 μL drops of TYH medium containing the OCCs to achieve a final sperm concentration of 1 × 10^5^/mL. The mixture drops were then incubated at 37°C under 5% CO_2_ for further capturing by the inverted microscope (Leica, Wetzlar, Germany) and assessment of fertilisation and embryo development rates.

### 2.15 Statistical Analysis

Data were expressed as mean ± SD, at least three independent tests were performed in each study, and similar results were acquired. Statistical analysis was performed using GraphPad Prism software (Version 9.0.1; GraphPad Software, United States). Levene's test was used to evaluate the homogeneity of the variance of the data. After testing the normality of distribution by Shapiro–Wilk test (Sample size < 50), Student's t‐test was used to compare differences between two groups. ANOVA and Fisher's least significant difference (LSD) test were used to compare differences among multiple groups. When *p*‐value < 0.05, the difference was considered to be statistically significant; *indicated *p*‐value < 0.05; **indicated *p*‐value < 0.01; ***indicated *p*‐value < 0.001; and ****indicated *p*‐value < 0.0001.

## 3 Results

### 3.1 *GADD45A* is Dysregulated in GCs of DOR Patients and is Crucial for Differentiation of Ovarian GCs

**FIGURE 1:**
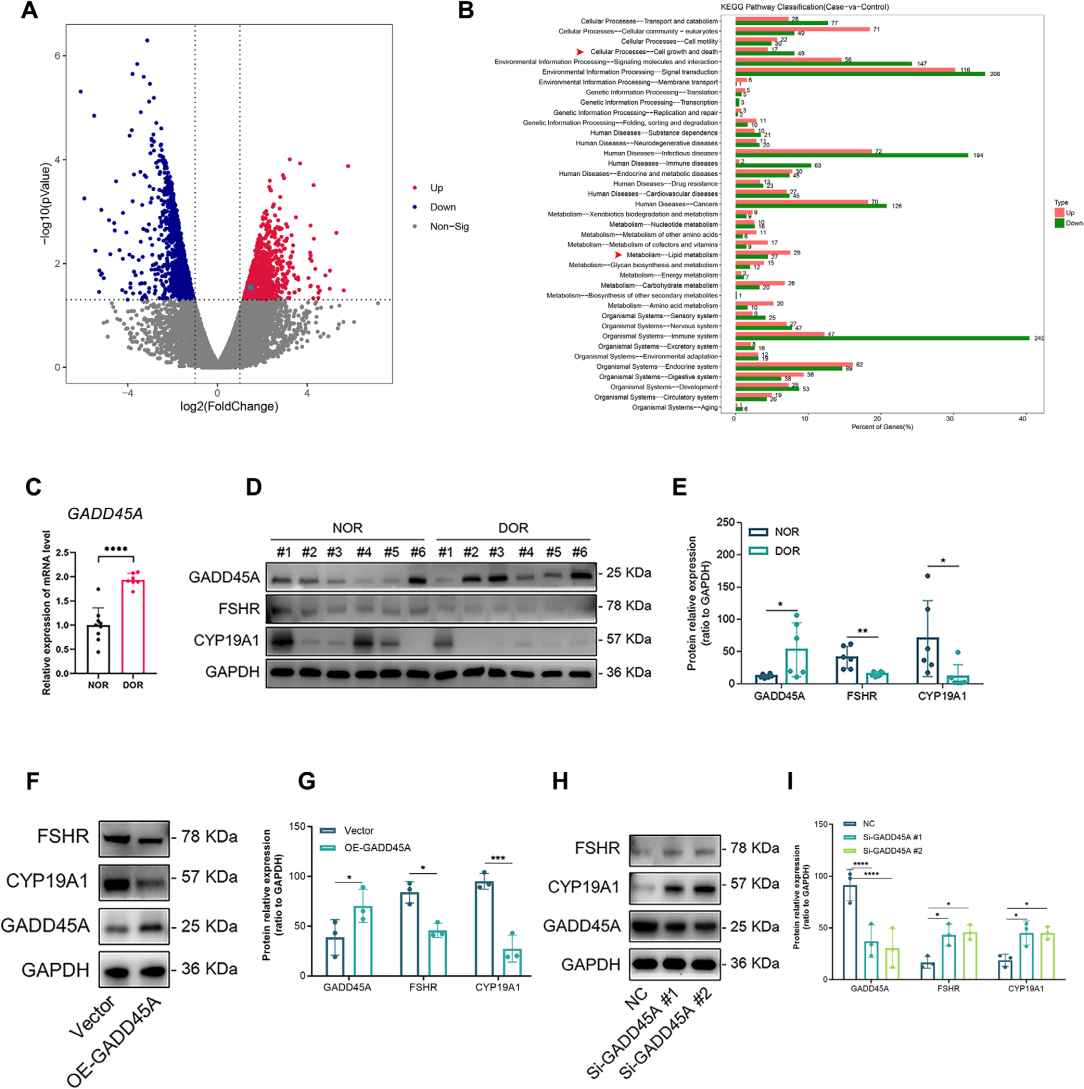
GADD45A is dysregulated in GCs of DOR patients and can regulate differentiation of ovarian GCs. (A) Volcanic map of RNA sequencing differentially expressed genes in the diminished ovary reserve (DOR) group, in which *GADD45A* is highlighted in upregulated genes compared with the Normal group (NOR). Three individual NOR samples and three individual DOR samples were used for RNA sequencing. (B) Differentially expressed genes for KEGG enrichment (*Q*‐value ≤ 0.05). Red arrowheads were used to mark two interested pathways. (C) The qPCR result showed *GADD45A* mRNA level in GCs of DOR group increased compared to NOR group. Nine individual NOR samples and seven individual DOR samples were used for qPCR examination. (D) Western Blots results revealed the elevated expression of GADD45A and reduced expression of FSHR and CYP19A in the GCs of DOR compared to NOR. Six individual NOR samples and six individual DOR samples were used for Western Blots. (E) The grey value analysis of the western blot (D) using its GAPDH density to normalise. (F) Western Blots results showed down‐regulated expression of FSHR and CYP19A when we overexpressed GADD45A in KGN cells. (G) The grey value analysis of the western blot (F) using its GAPDH density to normalise. (H) Western Blots results revealed that the expression of FSHR and CYP19A elevated when we knock downed the expression GADD45A in KGN cells using Si‐GADD45A#1 or Si‐GADD45A#2, comparing to control group. (I) The grey value analysis of the western blot (H) using its GAPDH density to normalise. Data are presented as the mean ± SD, *p < 0.05 compared to control, **p < 0.01 compared to control, ***p < 0.001 compared to control, ****p < 0.0001 compared to control.

Granulosa cells (GCs) from three control women with normal AMH/AFCs and three patients diagnosed with DOR who had decreased AMH/AFCs were included for transcriptome sequencing to find the differentially expressed genes. There is no difference in age, BMI and infertility duration between the two groups. Compared with control women, the AMH level (0.98 ± 0.36 vs. 4.02 ± 2.11 ng/mL, *p* < 0.0001) and the number of AFCs (4.33 ± 1.17 vs. 12.17 ± 4.45, *p* < 0.0001)were significantly decreased in DOR patients, while the FSH level (12.89 ± 2.26 vs. 6.59 ± 1.73, *p* < 0.0001) in DOR patients was obviously promoted (Table S1). In total, 2201 genes were detected in differentially expressed genes (Figure 1A). Intriguingly, we found *GADD45A* expression in GCs of DOR was 2.78‐fold higher in GCs of the control group. The differentially expressed genes were included in KEGG enrichment analysis shown in Figure 1B. The pathway Cell growth and death is related to the function of *GADD45A*. Correspondingly, we also observed the lagging cell growth in GCs of DOR compared with GCs of the control group when we cultured the cumulus granulosa cells after retrieval. Additionally, we noticed the enriched pathway lipid metabolism, which is indispensable for GC differentiation to maintain folliculogenesis. We continued to collect other GC samples (NOR *n* = 15, DOR *n* = 11) to verify the dysregulated *GADD45A* expression in pathological conditions. The clinical features of the participating control and DOR patients are presented in Table S1. The qPCR result showed the expression of *GADD45A* was significantly increased in GCs of DOR (Figure 1C). This result was repeatedly proved at the protein level by Western Blot (Figure 1D,E). Furthermore, we detected the marker genes of GC differentiation *FSHR* and *CYP19A1*, which are also essential for steroidogenesis. Noticeably, FSHR and CYP19A1 were obviously reduced in the GCs of DOR (Figure 1D,E), indicating that the differentiation of GCs in DOR was impaired along with the upregulated expression of GADD45A. To explore the association of *GADD45A*, *CYP19A1* and *FSHR*, we used the KGN cell line to overexpress and knock down GADD45A in vitro. When GADD45A was overexpressed in KGN, the protein levels of CYP19A1 and FSHR were decreased (Figure 1F,G). However, after we transfected siRNA to knock down *GADD45A* for 48 h in KGN, the alteration of CYP19A1 and FSHR presented opposite results in that CYP19A1 and FSHR were significantly upregulated (Figure 1H,I). Collectively, these results showed that the expression level of *GADD45A* can regulate the homeostasis of GC differentiation. The dysregulated *GADD45A* in GCs may account for the impaired differentiation of GCs in DOR to some extent. It was speculated that *GADD45A* was crucial for maintaining normal ovarian function.

### 3.2 *GADD45A* Overexpression Can Inhibit the GCs Proliferation but Knockdown Has no Effect

**FIGURE 2:**
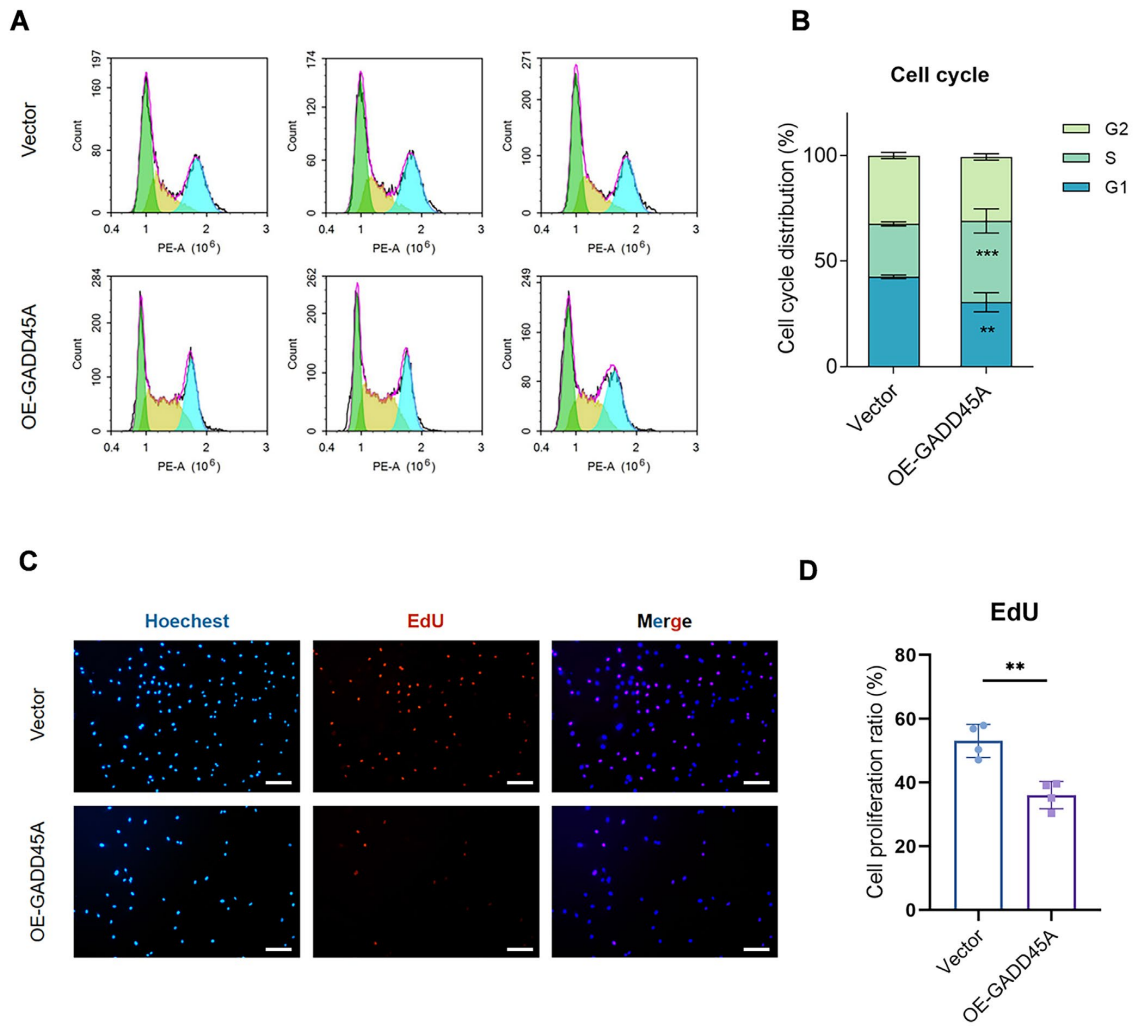
GADD45A overexpression could inhibit GCs proliferation. (A) The flow cytometry results showed the increased S‐phase and decreased G1‐phase distribution in the cell cycle of OE‐GADD45A KGN compared with control KGN in three replication assays. (B) The histogram manifested the corresponding statistical graphs of cell proportion at different cycle stages in OE‐GADD45A group and vector group (control). (C) EdU (red) was used to label proliferating KGN cells, and the nuclei were stained with Hoechst 33342 (blue) in Vector KGN (control) and OE‐GADD45A KGN cells. Scale bars, 100 μm. (D) Quantitative analysis showed that the EdU‐positive rates of the OE‐GADD45A group were much less than control. The data were repeated for four independent assays. Data are presented as the mean ± SD, **p < 0.01 compared to control, ***p < 0.001 compared to control.

To further understand the effect of abnormal expression of GADD45A in GCs, considering the canonical function of *GADD45A* in growth arrest, we used PI staining in *GADD45A* overexpressed KGN and analysed on flow cytometry. It was found that overexpression of *GADD45A* could lead to S‐phase cell cycle arrest as compared with control (Figure 2A,B). Additionally, we examined the proliferation of KGN cells after *GADD45A* overexpression using 5‐ethynyl‐2′‐deoxyuridine (EdU) staining. The fluorescence result showed a reduced proportion of EdU‐positive cells in OE*‐GADD45A* KGN (Figure 2C,D). We next investigated whether the *GADD45A* knockdown could lead to the contrary effect relative to overexpression. After using SiRNA to knock down *GADD45A*, we performed the cell cycle analysis, EdU staining and found there was no obvious difference in the growth of cultured KGN cells (Figure S1A–D). In sum, combined with previous results in the study, it was inferred that loss of GADD45A could only affect GCs differentiation side, while overexpression of GADD45A could impair both sides of GCs differentiation and proliferation.

### 3.3 *Gadd45a*‐Deficient Mouse Model Establishment and Determination of the Expression of *Gadd45a* in Ovary

**FIGURE 3:**
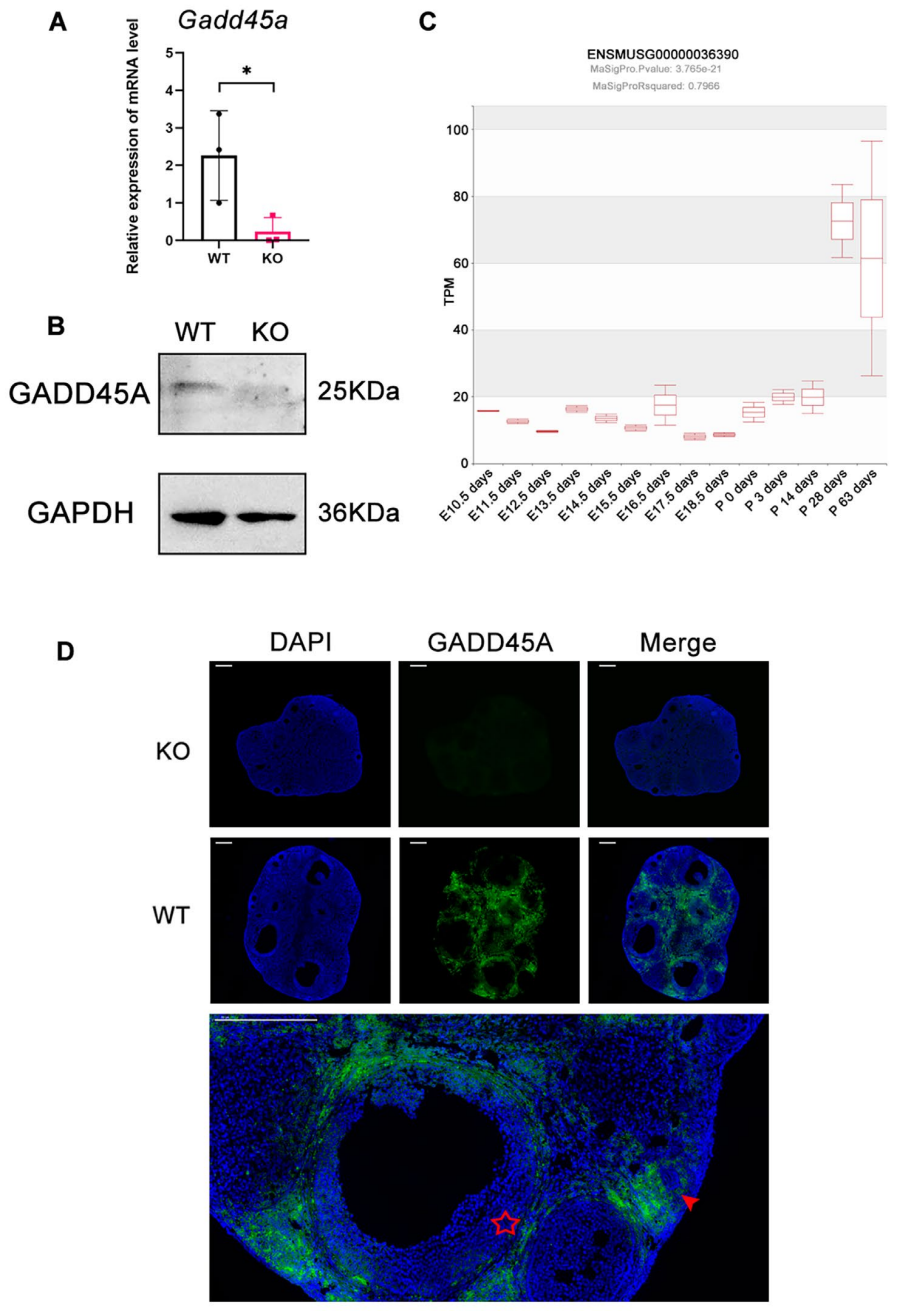
Expression of *Gadd45a* in mouse ovaries. (A) qPCR results showed ovaries of *Gadd45a* KO mice had much lower *Gadd45a* mRNA level compared with WT ovaries, which confirmed the knockout efficiency. (B) Western blot results confirmed the knockout of *Gadd45a* protein level in the KO ovary compared with WT ovary. (C) The RNA expression levels of *Gadd45a* at different developmental stages in mouse ovaries (TPM = transcripts per million; E = embryonic day, *P* = postnatal day). (D) Immunofluorescence of GADD45A expression in the ovaries of KO and WT mice. GADD45A is barely visible in the ovaries of *Gadd45a* KO mice and is robustly expressed in ovarian stroma and the granulosa cells of atretic follicles. We used the five‐pointed star to mark antral follicles and the arrow head to mark atretic follicles. The data were repeated for three independent assays. *p < 0.05 compared to control.

To further validate the function of *Gadd45a* on granulosa cell differentiation and ovarian reserve in vivo, we generated a *Gadd45a*‐deficient mouse model lacking exons 1–4 of *Gadd45a* using the CRISPR–Cas9 approach (Figure S2A). The PCR genotype identification of heterozygous F1 and WT mouse tails is shown in Figure S2B. The knockout of *Gadd45a* in the ovaries was confirmed by qPCR and Western blot analysis (Figure 3A,B). Then we explored the public database MeDAS [25] to find out the RNA expression levels of *Gadd45a* at different developmental stages in mouse ovaries. The data showed that *Gadd45a* expression rose to a high level since postnatal day 28, which is the important time point for mice ovary maturity [26]. The localization of GADD45A to specific compartments of the ovary and follicles at different stages was assessed by immunofluorescence. As expected, GADD45A was barely visible in *Gadd45a* KO mice ovaries. In WT mice, GADD45A was robustly expressed in ovarian stroma, granulosa cells of atretic follicles and barely expressed in the granulosa cells of the primordial follicles, primary follicles, secondary follicles and antral follicles (Figure 3D).

### 3.4 Impaired Estrous Cycling, Disturbed Ovarian Hormone Levels in Gadd45a KO Mice

**FIGURE 4:**
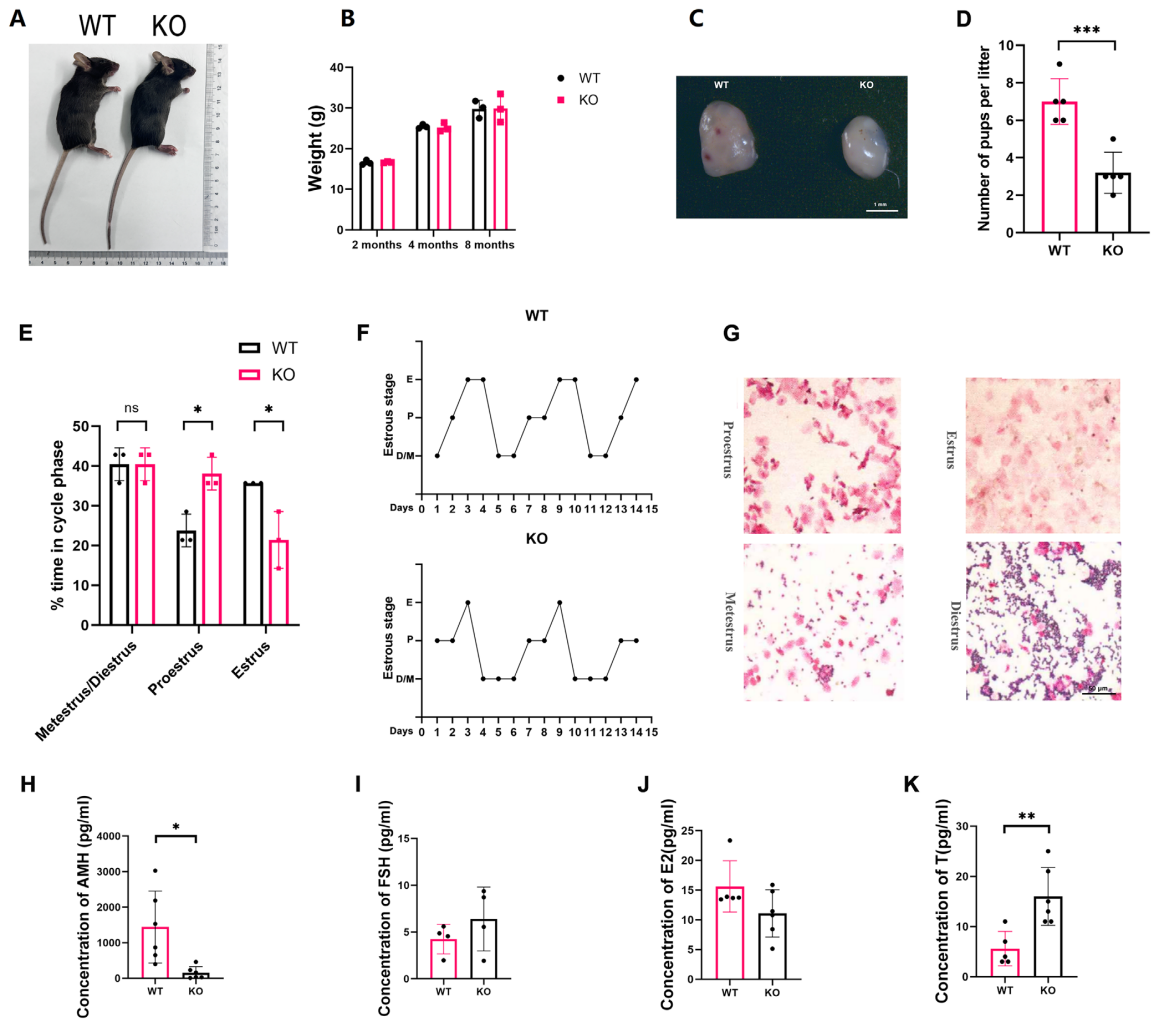
*Gadd45a* KO causes impaired estrous cycling and ovarian hormones disorder. (A) The appearance of mice, the body size of KO mice was comparable with WT mice. (B) The body weight of mice at the age of 2 months, 4 months and 8 months is comparable between WT and KO mice. (C) The appearance of mouse ovaries showed KO ovary is smaller than WT ovary. (D) Litter size of KO mice reduced compared to the WT group. *n* = 5, each group (E) The distribution result of oestrous cycle phases showed that the proestrus phase duration was significantly prolonged in *Gadd45a* KO mice compared to WT mice. Conversely, the oestrus phase was markedly shorter in *Gadd45a* KO mice than in WT mice. (F) Representative oestrous cyclicity of 3 mice/group during 14 consecutive days. M/D, metestrus/diestrus phase; P, proestrus; E, oestrus. (G) Representative images of oestrous cycle phases, proestrus, oestrus, metestrus and diestrus, respectively. (H) The reduced concentration of AMH in KO mice compared to WT mice. (I) The elevated but not significant concentration of FSH concentrations in KO mice compared to WT mice. (J) The reduced but not significantly E2 concentrations in KO mice compared to WT mice. (K) The elevated T concentrations in KO mice compared to WT mice. Data are presented as the mean ± SD, ns, not significant compared to WT mice, *p < 0.05 compared to WT mice, **p < 0.01 compared to WT mice, ***p < 0.001 compared to control.

*Gadd45a* KO mice were generated by natural conception from heterozygous parents. Then, heterozygous (+/−) female mice and male KO mice were caged together to produce more KO mice. To exclude potential developmental alterations that would influence ovarian function because of the whole‐body knockout, we recorded the body size of WT and KO mice at the same age (Figure 4A). We found that the body weight had no difference at the age of 2, 4, and 8 months in WT and KO mice (Figure 4B). Noticeably, ovaries from the *Gadd45a* KO mice were smaller than those from the WT mice (Figure 4C). We also examined the fertility of the *Gadd45a* knockout female mice by mating them with 9‐week‐aged male mice. We found that there was a decreased number of offspring in the KO mice group (Figure 4D). The frequency and duration of the estrous cycle reflect the hormonal milieu that maintains ovulatory function. Hence, we analysed the vaginal cytology of mice. Representative images of vaginal cytology for each oestrous cycle stage are shown in Figure 4G, and the proportion of mice in each stage of the estrous cycle was also assessed. The results showed that KO mice had more irregular cycles than regular cycles, where a complete oestrous cycle was defined in 4–5 days, and cycle proportions were present in WT mice. The proestrus time of KO mice was significantly prolonged compared to that of WT mice; however, oestrus was much shorter in Gadd45a KO mice than in WT mice (*p* < 0.05) (Figure 4E,F).

To evaluate ovarian function in *Gadd45a* KO mice, the serum levels of AMH, FSH, estradiol, and testosterone were determined by ELISA. Compared with that in WT mice, the AMH concentration in *Gadd45a* KO mice was significantly lower (154.7 ± 174.2 pg/mL vs. 1445 ± 1012 pg/mL, *p* < 0.05) (Figure 4H). The FSH concentration of KO serum was higher than the counterpart of WT, but the difference was not significant (6.393 ± 3.41 pg/mL vs. 4.25 ± 1.58 pg/mL, *p* = 0.29) (Figure 4I). The lower AMH level and higher FSH level suggested poor ovary function in the KO ovary. Additionally, the concentration of oestradiol had slightly reduced in KO serum but had no significance (11.09 ± 3.99 pg/mL vs. 15.62 ± 4.33 pg/mL, *p* = 0.10) (Figure 4J). Whereas, the concentration of testosterone in KO serum was elevated markedly as compared with WT (16.00 ± 5.76 pg/mL vs. 5.60 ± 3.44 pg/mL, *p* < 0.01) (Figure 4K). As the results in KGN (Figure 1), the in vivo data indicated that *Gadd45a* regulated the balance of GC differentiation, and these results of hormones probably resulted from the joint effect of GC homeostasis disruption and self‐gene compensations.

### 3.5 Reduced Ovarian Reserve in KO Mice and Partially 2‐Cell Block in IVF Outcome

**FIGURE 5:**
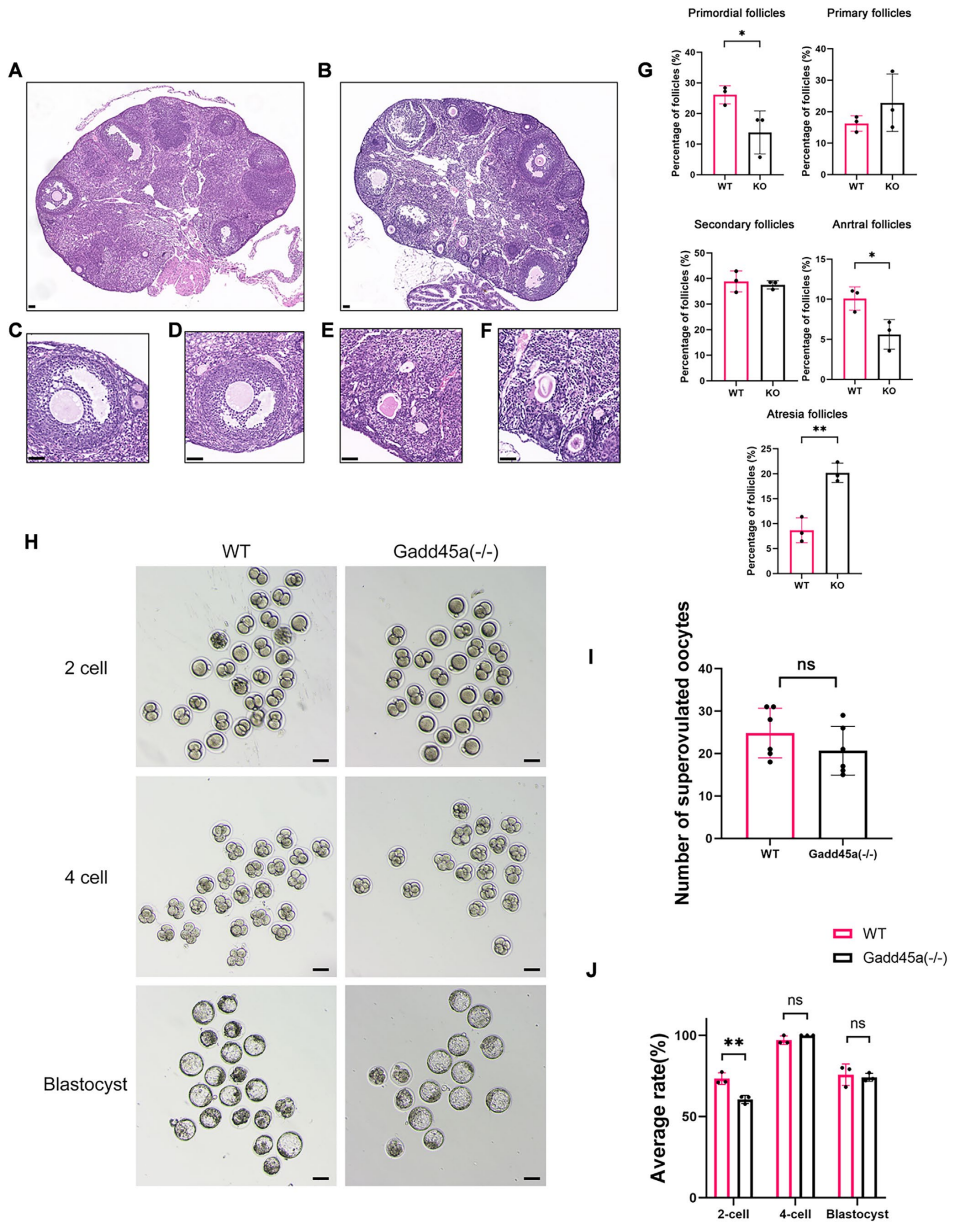
Reduced ovarian reserve in KO mice and poor‐quality oocytes in IVF outcome. (A) H&E staining of follicles in different stages in WT mice. (B) H&E staining of follicles in different stages in *Gadd45a* KO mice. (C) Representative image of typical primordial follicles, primary follicles, secondary follicles. (D) Representative image of typical antral follicles. (E, F) Representative image of typical atretic follicles. Scale bars, 50 μm (G) Percentage of primordial follicles, primary follicles, secondary follicles, antral follicles and atresia follicles. The percentages of primary follicles and secondary follicles are comparable between WT and KO mice. The percentage of primordial follicles and antral follicles in KO mice are less than the counterpart of WT mice. The proportion of atresia follicles in KO mice are more than WT mice. (H) The IVF outcome of WT group and KO group by mating with WT male mice. (I) The number of superovulated oocytes of WT mouse and KO mouse using exogenous hormones has no difference between WT and KO mice. (J) Statistics of embryo development rates revealed that 2‐cell percentage in KO group is slightly impaired compared to WT group (*n* = 3 biologically independent WT mice or KO mice, scale bars, 100 μm). Data are presented as the mean ± SD; *p < 0.05 compared to WT mice; **p < 0.01 compared to WT mice；ns, not significant.

Apparent histological changes were observed in the ovaries of *Gadd45a* KO mice (Figure 5A,B). Representative images of follicles in different stages are shown in Figure 5C–F. Compared to the follicles of WT mice, significant reduction was observed in the percentage of primordial follicles (13.81% vs. 26.1%, *p* < 0.05) and antral follicles (5.62% vs. 10.09%, *p* < 0.05) in *Gadd45a* KO mice. Moreover, the percentage of atretic follicles (20.19% vs. 8.66%, *p* < 0.05) was substantially increased in *Gadd45a* KO mice. No difference was found in primary follicles or secondary follicles (*p* > 0.05) (Figure 5G). To investigate the ability of ovulating and the outcome of IVF for KO mice using exogenous hormones, we injected 5 IU PMSG per mouse to superovulate on the first day and after 48 h injected 5 IU hCG per mouse. The number of superovulated oocytes was counted after fertilisation of oocyte‐cumulus complexes (OCCs). There is no difference between superovulated oocytes of WT mice and KO mice (Figure 5I). Then, we used sperm from WT male mice to perform IVF with superovulated eggs from both KO female mice and WT female mice. Intriguingly, the 2‐cell rate of *Gadd45a* KO oocytes was lower than that of WT while 4‐cell and blastocyst rates were comparable (Figure 5H,J). In conclusion, *Gadd45a* knockout could cause the diminished ovarian reserve but superovulation using exogenous hormones can temporarily ovulate enough eggs. The IVF results indicated that the oocytes from KO mice may have a potential poor quality. To exclude the possibility that *Gadd45a* itself may play a role in oocytes and that knocking out *Gadd45a* could directly affect oocyte quality, we utilised publicly available single‐cell transcriptome data (The Data was retrieved from the study [27]) to compare the expression levels of *Gadd45a* in oocytes and granulosa cells. We found that the expression level of *Gadd45a* in oocytes is significantly lower than in granulosa cells, which indicated the gene is mainly functioning in granulosa cells (Figure S3A). Therefore, the potential poor quality of oocytes might result from the aberrant interactions between GCs and oocytes.

## 4 Discussion

DOR is one of the most challenging diseases in reproductive medicine. Multiple mechanisms have been found to be causative of DOR, including the presence of abnormal follicle stock formation, increased follicular atresia, impaired recruitment of dominant follicles, blocked follicular maturation or rapid depletion of the follicular stock [3, 6, 7]. This study is trying to figure out a novel etiological factor in DOR pathogenesis.

Our study reveals that GADD45A is upregulated in granulosa cells of DOR patients, inversely correlating with reduced FSHR and CYP19A1 expression. Functional studies in KGN cells demonstrated that GADD45A suppresses GC differentiation by regulating these genes. *Gadd45a* knockout mice recapitulated DOR phenotypes, including endocrine dysfunction, diminished AMH levels, reduced primordial/antral follicles, increased follicular atresia and poor oocyte quality. Collectively, these findings establish GADD45A as a key regulator of ovarian reserve and fertility, positioning *Gadd45a* KO mice as a novel DOR model and highlighting *GADD45A* as a therapeutic target.

Our findings in mice and humans are similar to the phenotype in other species and relevant models mentioned by previous studies. Costermans et al. [15] found that GADD45A abundance was lower in sows with a high percentage of high‐quality cumulus‐oocyte complexes and that it could thus serve as a marker of follicle quality. Furthermore, the abundance of GADD45A is higher in porcine atretic follicles and might provide a valuable marker for early atretic follicles [16]. GADD45A expression in bovine cumulus cells is increased when cumulus cells are cultured with oocytes, indicating that GADD45A participates in the bidirectional communication between the oocyte and its surrounding cumulus cells [17]. However, GADD45A is overexpressed in PCOS, a condition in which folliculogenesis is arrested and AMH is elevated [28]. GADD45A plays a protective role in normal cells which is mediated via a complex interplay of physical interactions with other cellular proteins implicating in cell cycle regulation and the cellular stress response, notably proliferating cell nuclear antigen (PCNA), cdnk1a, cdc2/cyclinB1 and the p38 and JNK stress response kinases in normal development and tumorigenesis [9, 11, 29]. Several previous studies supported that the MAPK signalling pathway in granulosa cells promotes oocyte development. A key study indicates that the MAPK signalling pathway in granulosa cells mediates the main physiological functions of LH and plays an important role in ovulation, follicle arrest and corpus luteum formation. *ERK1/2* knockout mice in granulosa cells have defects in LH‐induced oocyte meiotic resumption, ovulation and luteinization [30]. What is more, we found that the negative correlation between the level of GADD45A and the expression of CYP19A1 and FSHR indicates that GADD45A inhibits the differentiation of GCs by regulating the expression of its downstream target genes. The loss of *Gadd45a* in mice caused aberrant follicle development and endocrine disorder. Perhaps because of the premature differentiation of mouse GCs due to the upregulated expression of FSHR and CYP19A1, leading to aberrant follicle development. Other studies have reported other mouse models with similar phenotypes because of premature GCs differentiation. In vitro inhibition of *Wt1* in granulosa cells could stimulate *FSHR*, *3β‐HSD* and *CYP19A1*. The mutant *Wt1* mice exhibited ovarian follicle development defects which were similar to premature ovarian failure [31]. The decreased oestrogen level and aberrant testosterone level may result from reduced number of antral follicles and endocrine homeostasis disruption. All these studies indicate that GADD45A is a critical regulator of ovarian follicular development, and that dysregulation is involved in follicle‐related diseases such as DOR, POF and PCOS.

This study introduces novel insights into the molecular pathogenesis of diminished ovarian reserve by establishing GADD45A as a novel regulator of granulosa cell function and ovarian reserve dynamics. To our knowledge, this is the first investigation of GADD45A upregulation in GCs of DOR patients. Notably, our findings reveal a dual regulatory role of GADD45A in GC biology: while its overexpression suppresses both proliferation and differentiation, its knockdown selectively enhances granulosa cell differentiation markers (e.g., CYP19A1, FSHR), suggesting context‐dependent roles in follicular development. GADD45A dysregulation disrupts GC differentiation trajectories while accelerating follicle attrition. The generation and characterisation of the *Gadd45a* knockout mouse model constitute a major methodological innovation. These animals faithfully recapitulate clinical DOR hallmarks—including primordial follicle depletion, hormonal dysregulation and impaired oocyte developmental competence—enriching a genetically defined platform for studying DOR progression.

Resting primordial follicles comprise the “ovarian reserve”, the size of which is a critical indicator of female fertility and the approximate determinant of the reproductive lifespan [1, 10]. AMH is widely applied to the diagnosis of DOR and was found to be severely reduced in *Gadd45a* KO mice. AMH was mostly present in the granulosa cells of secondary, preantral and small antral follicles ≤ 4 mm in diameter in human and mice [32]. The percentage of antral follicles in WT mice is significantly higher than in KO mice. Although the exact functions of AMH in folliculogenesis are unclear, multiple studies have demonstrated the positive effect of AMH on follicle growth [33]. AMH is secreted by the granular cells of growing follicles at primary stages and provides negative feedback on primordial follicle activation to preserve the primordial follicle pool, potentially sparing the germ cell [33, 34]. Limiting primordial follicle activation by adding exogenous AMH could lessen the premature depletion of the ovarian reserve [35, 36]. The excessive activation of primordial follicles in Gadd45a KO mice was seen through a degree of increasing trend of primary follicles. When the growing follicles are converted to atretic follicles in *Gadd45a* KO mice, the negative feedback occurs and the decreased level of AMH in serum might over‐activate primordial follicles, causing them to become growing follicles, thus contributing, at least partially, to the premature depletion of the primordial follicle pool with ovarian volume shrinkage [5, 6, 35, 37].

This work opens new avenues for therapeutic strategies targeting granulosa cell differentiation to preserve ovarian reserve and improve the diagnostic framework by proposing GADD45A as a potential biomarker in granulosa cells for DOR risk stratification. The established *Gadd45a* mouse model will serve as an indispensable tool for evaluating interventions aimed at mitigating ovarian aging, with translational implications for fertility preservation.

As the limitations of the present study, we have not explored the specific mechanism underlying the relatively poor quality of oocytes in *Gadd45a* KO mice. Follicular development is a complex and highly coordinated process that is finely regulated by many factors, including the growth of oocytes and the proliferation and differentiation of granulosa cells, as well as the ovarian microenvironment [38, 39]. Natriuretic Peptide C (NPPC) produced in mural granulosa cells (MGCs) can activate receptors on MGCs and cumulus cells, Natriuretic Peptide Receptor 2 (NPR2), which can generate cGMP [40, 41]. The cGMP in cumulus cells and granulosa cells can diffuse to oocytes through gap junctions and inhibit the activity of Phosphodiesterase 3A (PDE3A) in oocytes, thereby inhibiting cAMP hydrolysis [42, 43, 44, 45, 46] and maintaining high levels of cAMP, activating protein kinase A (PKA), causing CDK to be phosphorylated and inactivated and blocking oocytes at the prophase of meiosis [44, 45, 46]. Therefore, the interaction of oocytes and granulosa cells is crucial for folliculogenesis. The poor quality of oocytes in *Gadd45a* KO mice was found by the decreased 2‐cell rate of early embryos in vitro fertilisation. The underlying mechanism we suppose may be the adverse impact by GCs through communication between GCs and oocytes while a relatively low degree of GADD45A expression in the oocytes themselves may serve a function. The conditional knockout of *Gadd45a* in GCs or oocytes should be further investigated. Furthermore, it was reported that GADD45A has several epigenetic roles to regulate downstream genes. GADD45A can act as an epigenetic regulator to promote DNA demethylation via TET1 [47, 48]. Moreover, GADD45A is also an RNA‐binding protein (RBP) which could maintain the stability of mRNAs via a transcription‐independent mechanism [49]. Therefore, the potential molecular mechanism of GADD45A to regulate FSHR and CYP19A1 is valuable to be further explored.

In conclusion, we proposed that the dysregulation of *GADD45A*, either upregulation or down‐regulation in granulosa cells, may account for the aetiology of DOR. GADD45A might participate in the regulation of ovarian follicle development and target granulosa cell function through cell differentiation and ovarian microenvironment homeostasis. Our findings could provide Gadd45a KO mice as an ideal DOR mouse model for further mechanistic investigation, and GADD45A expression in granulosa cells may be a sensitive biomarker in DOR patients. More detailed molecular biological experiments using specific cell types are warranted to thoroughly understand the role of GADD45A in the etiological study of DOR.

## Author Contributions

**Juncen Guo:** data curation (equal), formal analysis (equal), investigation (equal), methodology (equal), software (equal), validation (equal), visualization (equal), writing – original draft (equal), writing – review and editing (equal). **Yuanyuan Hu:** data curation (equal), formal analysis (equal), investigation (equal), methodology (equal), software (equal), validation (equal), visualization (equal), writing – original draft (equal), writing – review and editing (equal). **Qi Cao:** data curation (equal), formal analysis (equal), investigation (equal), methodology (equal), resources (equal), software (equal), validation (equal), visualization (equal). **Ying Zhang:** data curation (equal), formal analysis (equal), investigation (equal), methodology (equal), software (equal), supervision (equal), validation (equal), visualization (equal), writing – review and editing (equal). **Yihe Jia:** investigation (equal), methodology (equal), supervision (equal), validation (equal). **Lan Liu:** data curation (equal), methodology (equal), validation (equal), visualization (equal). **Yanru Zeng:** data curation (equal), formal analysis (equal), validation (equal), visualization (equal). **Xiao Wu:** data curation (equal), formal analysis (equal), software (equal), validation (equal). **Yuelin Song:** formal analysis (equal), software (equal), visualization (equal). **Maosen Yang:** data curation (equal), investigation (equal), software (equal), supervision (equal). **Wenming Xu:** conceptualization (equal), funding acquisition (equal), project administration (equal), resources (equal), writing – review and editing (equal). **Yang Hu:** data curation (equal), formal analysis (equal), methodology (equal), resources (equal), supervision (equal), validation (equal). **Wei Huang:** conceptualization (equal), data curation (equal), project administration (equal), resources (equal), supervision (equal). **Tian Tang:** conceptualization (equal), data curation (equal), funding acquisition (equal), investigation (equal), project administration (equal), resources (equal), supervision (equal), writing – original draft (equal), writing – review and editing (equal).

## Disclosure

The authors have nothing to report.

## Conflicts of Interest

The authors declare no conflicts of interest.

## Supporting information


**Figure S1:** *GADD45A* knockdown could not alter GCs proliferation. (A) The results showed the comparable cell cycle among NC group (control), Si‐GADD45A#1 and Si‐GADD45A#2 group detected by flow cytometry with three replication assays. (B) The histogram manifested the corresponding statistical graphs of cell proportion at different cycle stages in Si‐GADD45A#1 group, Si‐GADD45A#2 group and NC group. There is no difference in cell proportions among these groups. (C) EdU (red) was used to label proliferating KGN cells, and the nuclei were stained with Hoechst 33342 (blue). This fluorescence results showed that the EdU‐positive rates of three groups had no significant difference in Si‐GADD45A#1 group, Si‐GADD45A#2 group and NC group. The data are repeated for three independent assays. Scale bars, 100 μm. (D) Quantitative analysis showed that the EdU‐positive rates of the Si‐GADD45A group #1 and #2 have no difference compared to NC group. The data are repeated for three independent assays. ns, not significant.

**Figure S2:** Generation of *Gadd45a* KO mice. (A) Schematic representation of the gene targeting procedure, C57BL/6 *Gadd45a*‐heterozygous (+/−) female mice were constructed by CRISPR/Cas9 with deletion of a 2007‐bp fragment encompassing exons 1–4. (B) Confirmation of heterozygous *Gadd45a* mice, using WT mice as the negative control and nuclease‐free water as blank control (NC).

**Figure S3:** *Gadd45a* preferred to express in granulosa cells than oocytes. (A) The gene expression bubble plot from single‐sequencing of mice ovaries demonstrated that the expression of *Gadd45a* is more robust in granulosa cells compared to oocytes. *Amh* is the specific marker gene for granulosa cells which contain cluster A and cluster B. *Zp3* is the specific marker gene for oocytes. This result was analysed by an interactive web application. (https://omrf.shinyapps.io/OvarianAgingSCAtlas/).

**Table S1:** Characteristic of included DOR patients and control groups.
**Table S2:** Sequences of various siRNAs.

## Data Availability

The datasets generated and/or analysed during the current study are not publicly available but are available from the corresponding author on reasonable request.
