# Supplementary figures and images for: GADD45A is Essential for Granulosa Cells Differentiation and Ovarian Reserve in Human and Mice
Source: J Cell Mol Med. 2025 Sep 7;29(17):e70820. doi: 10.1111/jcmm.70820 (PMC12414809; doi:10.1111/jcmm.70820)

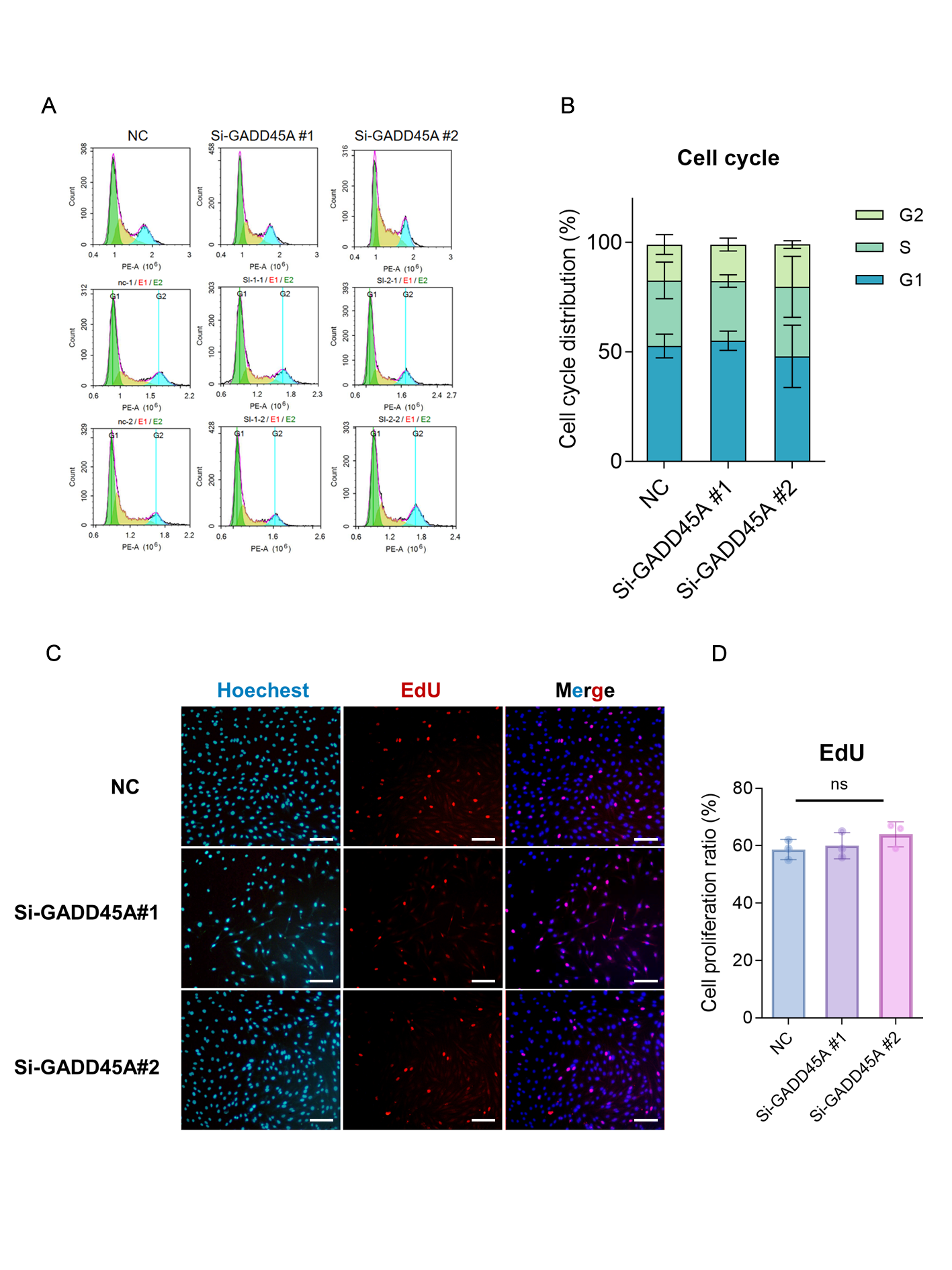

Supplement: Supplementary file 1 — Figure S1: GADD45A knockdown could not alter GCs proliferation. (A) The results showed the comparable cell cycle among NC group (control), Si‐GADD45A#1 and Si‐GADD45A#2 group detected by flow cytometry with three replication assays. (B) The histogram manifested the corresponding statistical graphs of cell proportion at different cycle stages in Si‐GADD45A#1 group, Si‐GADD45A#2 group and NC group. There is no difference in cell proportions among these groups. (C) EdU (red) was used to label proliferating KGN cells, and the nuclei were stained with Hoechst 33342 (blue). This fluorescence results showed that the EdU‐positive rates of three groups had no significant difference in Si‐GADD45A#1 group, Si‐GADD45A#2 group and NC group. The data are repeated for three independent assays. Scale bars, 100 μm. (D) Quantitative analysis showed that the EdU‐positive rates of the Si‐GADD45A group #1 and #2 have no difference compared to NC group. The data are repeated for three independent assays. ns, not significant. [file JCMM-29-e70820-s004.tif]

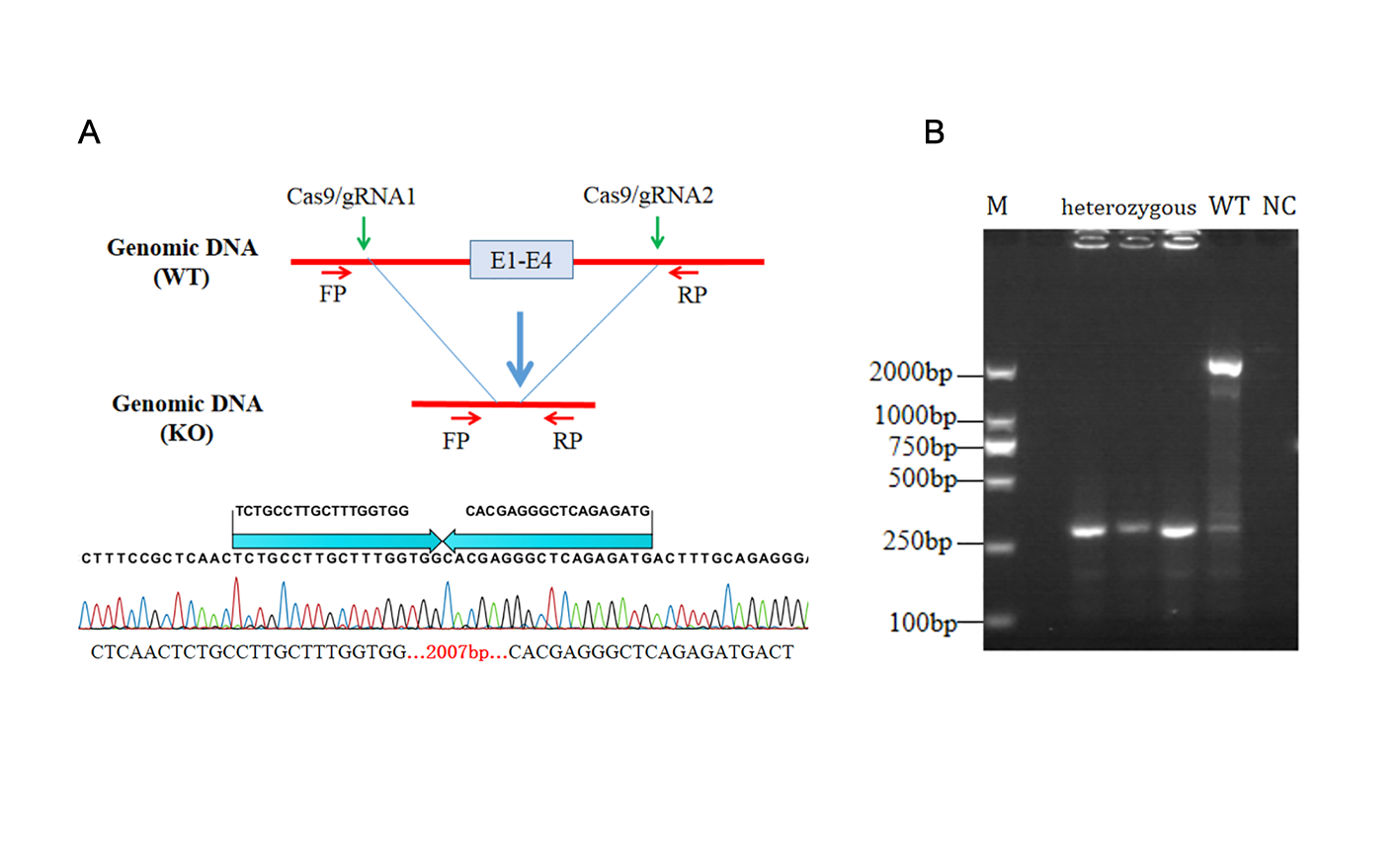

Supplement: Supplementary file 2 — Figure S2: Generation of Gadd45a KO mice. (A) Schematic representation of the gene targeting procedure, C57BL/6 Gadd45a‐heterozygous (+/−) female mice were constructed by CRISPR/Cas9 with deletion of a 2007‐bp fragment encompassing exons 1–4. (B) Confirmation of heterozygous Gadd45a mice, using WT mice as the negative control and nuclease‐free water as blank control (NC). [file JCMM-29-e70820-s001.tif]

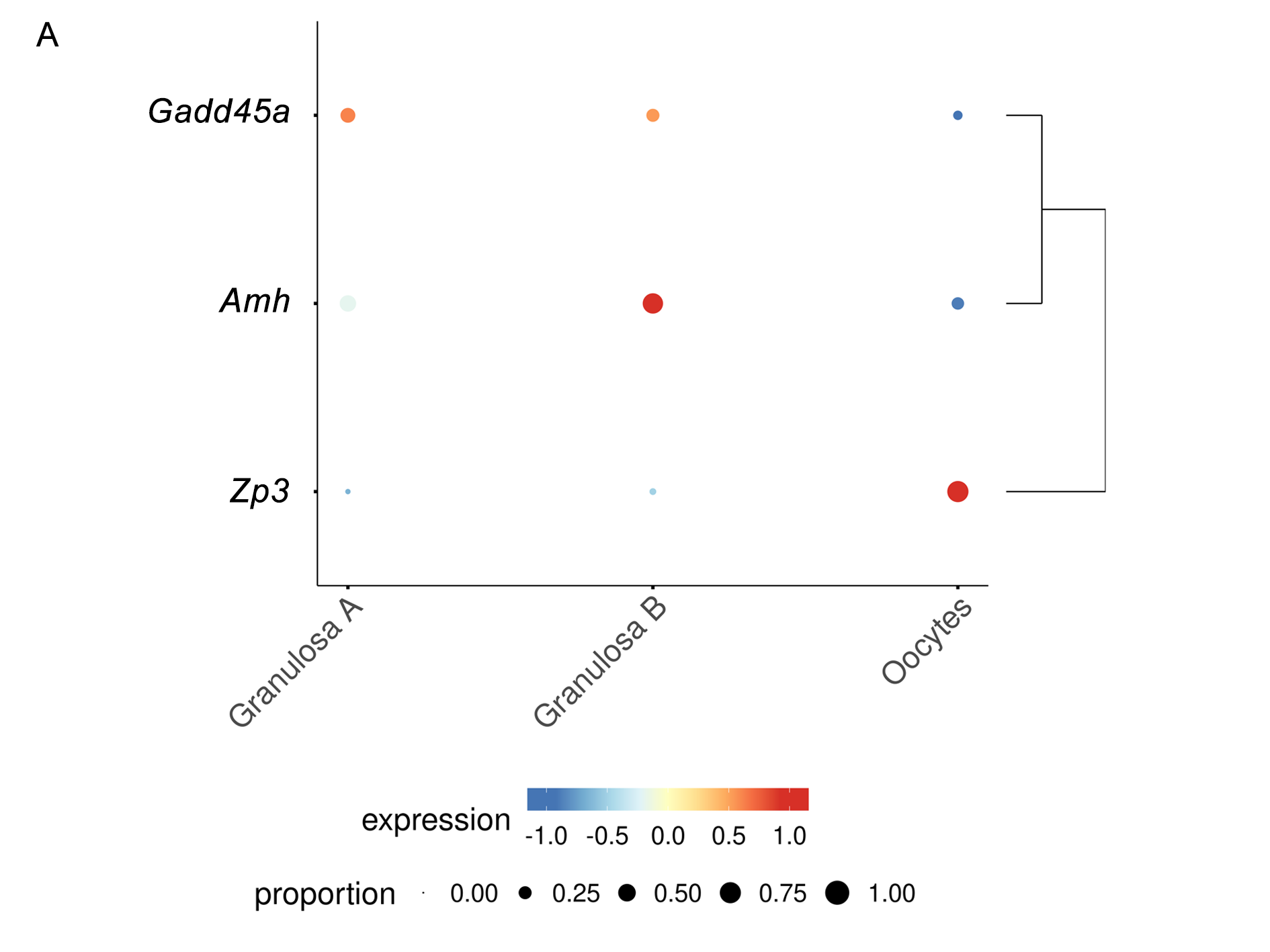

Supplement: Supplementary file 3 — Figure S3: Gadd45a preferred to express in granulosa cells than oocytes. (A) The gene expression bubble plot from single‐sequencing of mice ovaries demonstrated that the expression of Gadd45a is more robust in granulosa cells compared to oocytes. Amh is the specific marker gene for granulosa cells which contain cluster A and cluster B. Zp3 is the specific marker gene for oocytes. This result was analysed by an interactive web application. (https://omrf.shinyapps.io/OvarianAgingSCAtlas/). [file JCMM-29-e70820-s002.tif]
