# Supplementary material for: GADD45A is Essential for Granulosa Cells Differentiation and Ovarian Reserve in Human and Mice
Source: J Cell Mol Med. 2025 Sep 7;29(17):e70820. doi: 10.1111/jcmm.70820 (PMC12414809; doi:10.1111/jcmm.70820)
Supplement: Supplementary file 4 — Table S1: Characteristic of included DOR patients and control groups. Table S2: Sequences of various siRNAs. [file JCMM-29-e70820-s003.docx]

**Table S1 Characteristic of included DOR patients and control groups.**

| **RNA-Seq involved** | **NOR (n=3)** | **DOR (n=3)** | **P value** |
| --- | --- | --- | --- |
| **Age** | 31.35±3.24 | 32.71±3.17 | 0.075 |
| **BMI（kg/m2）** | 22.18±1.21 | 21.87±1.16 | 0.513 |
| **Years of infertility** | 4.71±2.14 | 3.98±2.35 | 0.019 |
| **FSH(mIU/ml)** | 6.59±1.73 | 12.89±2.26 | ＜0.0001 |
| **AMH(ng/ml)** | 4.02±2.11 | 0.98±0.36 | ＜0.0001 |
| **AFC** | 12.17±4.45 | 4.33±1.17 | ＜0.0001 |
| **qPCR involved** | **NOR (n=9)** | **DOR (n=7)** | **P value** |
| **Age** | 31.33 ± 2.42 | 30.67 ± 2.07 | 0.619 |
| **BMI (kg/m2)** | 21.81 ± 2.59 | 20.79 ± 2.07 | 0.469 |
| **Years of infertility** | 2.67 ± 1.51 | 1.83 ± 1.21 | 0.316 |
| **FSH (mIU/m))** | 7.04 ± 1.41 | 12.26 ± 4.26 | 0.017 |
| **AMH (ng/ml)** | 4.15 ± 0.95 | 0.67 ± 0.46 | < 0.0001 |
| **AFC** | 15.83 ± 4.54 | 4.17 ± 2.40 | 0.0002 |
| **WB involved** | **NOR (n=6)** | **DOR (n=6)** | **P value** |
| **Age** | 30.78 ± 1.64 | 32.71 ± 2.43 | 0.078 |
| **BMI (kg/m2)** | 20.05 ± 1.37 | 21.62 ± 3.36 | 0.221 |
| **Years of infertility** | 1.39 ± 0.60 | 1.86 ± 1.55 | 0.417 |
| **FSH (mIU/m))** | 6.41 ± 1.76 | 11.21 ± 5.00 | 0.017 |
| **AMH (ng/ml)** | 3.15 ± 1.16 | 0.64 ± 0.39 | < 0.0001 |
| **AFC** | 10.56 ± 2.07 | 3.86 ± 2.48 | < 0.0001 |

AFC. antral follicular count; AMH, low anti-Müllerian hormone; FSH, follicle stimulating hormone.

| **Gene** | **Sense (5'-3')** | **Antisense (5'-3')** |
| --- | --- | --- |
| Si-GADD45A#1 | GGAGAGCAGAAGACCGAAAGGTT | CCUUUCGGUCUUCUGCUCUCCTT |
| Si GADD45A#2 | GGAUCCUGCCUUAAGUCAACUTT | AGUUGACUUAAGGCAGGAUCCTT |
| Si-NC | UUCUCCGAACGUGUCACGUTT | ACGUGACACGUUCGGAGAATT |

**Table S2. Sequences of various siRNAs.**
